# *Escherichia coli* Exopolysaccharides Induced by Ceftriaxone Regulated Human Gut Microbiota *in vitro*

**DOI:** 10.3389/fmicb.2021.634204

**Published:** 2021-02-16

**Authors:** Baiyuan Li, Huahai Chen, Linyan Cao, Yunfei Hu, Dan Chen, Yeshi Yin

**Affiliations:** ^1^Key Laboratory of Comprehensive Utilization of Advantage Plants Resources in Hunan South, College of Chemistry and Bioengineering, Hunan University of Science and Engineering, Yongzhou, China; ^2^State Key Laboratory of Breeding Base for Zhejiang Sustainable Pest and Disease Control, Institute of Plant Protection and Microbiology, Zhejiang Academy of Agricultural Sciences, Hangzhou, China

**Keywords:** EPS-m2, ceftriaxone, gut microbiota, 16S rRNA gene sequencing, SCFA

## Abstract

A stable intestinal microflora is an essential prerequisite for human health. This study investigated the interaction between *Escherichia coli* exopolysaccharides (named EPS-m2) and the human gut microbiota (HGM) *in vitro*. The EPS-m2 was produced by *E*. *coli* WM3064 when treated with ceftriaxone. The monosaccharide composition analysis revealed that EPS-m2 is composed of glucuronic acid, glucose, fucose, galactose/N-acetyl glucosamine, arabinose, xylose, and ribose with a molar ratio of approximately 77:44:29:28:2:1:1. The carbohydrates, protein, and uronic acids contents in EPS-m2 was 78.6 ± 0.1%, 4.38 ± 0.11%, and 3.86 ± 0.09%, respectively. I*n vitro* batch fermentation experiments showed that 77% of EPS-m2 could be degraded by human fecal microbiota after 72 h of fermentation. In reverse, 16S rRNA gene sequencing analysis showed that EPS-m2 increased the abundance of *Alistipes*, *Acinetobacter*, *Alloprevotella*, *Howardella*, and *Oxalobacter*; GC detection illustrated that EPS-m2 enhanced the production of SCFAs. These findings indicated that EPS-m2 supplementation could regulate the HGM and might facilitate modulation of human health.

## Introduction

The human gastrointestinal tract carries a structured microbial composition that contributes to host nutrition, metabolism, and immunity; and the number of gut bacteria was approximately equal to the human cells (Sender et al., 2016). Increasing evidence suggests that microbial communities in the human gut have alterations in composition linked to dysbiosis-related diseases, such as various chronic diseases (Clemente et al., 2012; Rajilić-Stojanović et al., 2015). In recent years, significant interest has focused on the gut microbiota, which multiple factors, such as genetics, diet, and the environment contribute to the establishment of its ecosystem (Sommer and Backhed, 2013). Extensive investigations have found that diet plays a key role in the modulation of intestinal microbiota composition and metabolism (Zeevi et al., 2015; Adamberg et al., 2020). The gut microbiota involve increased energy harvesting and storage, and modulated various functions of host metabolism, such as assimilating undigested carbohydrates (Gill et al., 2006), a trait that probably has ecological and evolutionary forces that contribute to the establishment of a mutually beneficial relationship with human beings. There has been growing evidence that microbial metabolites exert an effect on the host’s physiology, namely, the immune system, and are involved in chemical interactions and signaling pathways (Hooper et al., 2012).

Carbohydrates comprise the essential fraction of our daily diet (Sonnenburg and Sonnenburg, 2014). Moreover, the maintenance and survival of the gut microbiota rely on the ability to assimilate dietary carbohydrates, which serve as the primary carbon and energy sources (Sonnenburg and Sonnenburg, 2014). The assimilation of carbohydrates can also change the composition of the gut microbiota, so scientists are developing carbohydrates as an alternative strategy to treat diseases by regulating the human gut microbiota (HGM). Exopolysaccharides (EPSs) are carbohydrate polymers that are synthesized and released outside of the bacterial cell wall. Microbial polysaccharides have attracted considerable attention and are exploited in food, cosmetic, and pharmaceutical industries (Chen et al., 2016; Silva et al., 2019). Especially, EPS produced by lactic acid bacteria (LAB) have wide diversity of structures without health risk and utilized in the field of food and pharmaceutical industries (Badel et al., 2011). However, the exact mechanisms of EPSs synthesis at genomic level are still limited. *Escherichia coli* are one of the world’s best-characterized organisms, and *E*. *coli* produced EPS also have gained attractive attention. *E*. *coli* secreted colanic acid (CA) has a positive effect on host lifespan of *Caenorhabditis elegans* by regulates mitochondrial dynamics and unfolded protein response (UPRmt) in the host (Han et al., 2017). A previous report also showed that *Escherichia coli* EPSs are critical for enhancing their adherence and resistance properties and also promoting the development of host immune system (Hufnagel et al., 2015). Moreover, our previous studies have confirmed that *E*. *coli* EC100 produced EPS-RB regulates the HGM and increases the abundance of SCFAs (Li et al., 2020). These results indicated that *E*. *coli* EPSs are similar to prebiotics and effective in the regulation of gut microbiota *in vitro* (Serafini et al., 2014). Thus, it is essential to understand the interactions between new polysaccharides with HGM.

Here, we isolated and characterized an EPS-m2, which was induced and produced in *E*. *coli* WM3064 when treated with ceftriaxone. Monosaccharide composition analysis illustrated that EPS-m2 is different from previously reported *E*. *coli* exopolysaccharides. *In vitro* studies established that human fecal microbiota could ferment the EPS-m2, and, in reverse, EPS-m2 can modulate human fecal microbiota *in vitro*. EPS-m2 fermentation also enhanced the SCFAs production.

## Materials and Methods

### Production and Preparation of *E*. *coli* EPS-m2

Culture media of Luria broth (LB) was used for *E*. *coli* growth and EPS-m2 production. DAP (2,6-diamino-pimelic acid) was added when culturing the *E*. *coli* WM3064 (*thrB1004 pro thi rpsL hsdS lacZ*ΔM15 *RP4-1360* Δ(*araBAD*) *567* Δ*dapA1341*::[*erm pir*(wt)]) strain that auxotrophic for DAP (Dehio and Meyer, 1997). The yields of EPS-m2, when treated with different antibiotic agents [ceftriaxone (CTRX), ampicillin (AMP), and imipenem (IMP)], were investigated. The antibiotic agents that generated the maximum output of EPS-m2 were used in further experiments. The *E*. *coli* WM3064 was inoculated in LB plates with 0.5 μg/mL ceftriaxone and 0.3 mM diaminopimelic acid at 37°C for 24–48 h. The cultures were scraped from the above plates and blended with phosphate-buffered saline (PBS). The EPS-m2 production was measured using the carbazole assay as previously described (Knutson and Jeanes, 1968). Briefly, the OD_600_ of the bacterial suspension in PBS, which corresponds to bacterial density, was measured. Cell pellets were removed by centrifugation at 12,000 g for 10 min, and the culture supernatant (700 μl) was mixed with 6 ml of borate-sulfuric acid reagent (10 mM H_3_BO_3_ in concentrated H_2_SO_4_) and 200 μl of 0.1% carbazole reagent. Following incubation at 100°C for 10 min and subsequent cooling, and absorbance was measured at 530 nm in a spectrophotometer. The relative yields of EPS-m2 were determined according to the ratio of OD_530_/OD_600_. For EPS-m2 preparation, the bacterial cultures were centrifuged at 12,000 g for 10 min and the resulting supernatant was filtered. Then, the supernatants were combined with three equal volumes of cold absolute ethanol overnight at 4°C. EPS-m2 was collected after centrifugation at 8,000 g for 20 min at 4°C and freeze-dried into powder for storage at -80°C until analysis.

### EPS-m2 Analyses

The anthrone-sulfuric acid method was used to measure the content of total polysaccharides of EPS-m2 (Roe, 1955). The protein contents in EPS-m2 were measured using Bradford’s method (Bradford, 1976). Uronic acid contents were determined according to carbazole colorimetry assay (Bitter and Muir, 1962). Glucose, bovine serum albumin and D-glucuronic acid were used as the standard, respectively. The monosaccharide composition of the crude EPS-m2 was evaluated as described by Li et al. (2020). All of the monosaccharide standards used in this study was purchased from YuanYe Bio-Technology Co., Ltd. (Shanghai, China).

### Fecal Sampling

A total of six healthy volunteers (labeled No. 1, No. 2, No. 3, No. 4, No. 5, and No. 6) living in China, aged 23–38 years, were selected for this study. Detailed information regarding these participants was included in Supplementary Table S1. All participants had not taken any antibiotic and not received prebiotic supplements in the past 3 months before sample collection. The study protocol was approved by the Ethics Committee of the Hunan University of Science and Engineering and Zhejiang Academy of Agricultural Sciences. Fresh fecal samples were immediately collected after defecation. A portion of each sample was homogenized by vortexing in 0.1 M pre-reduced PBS (pH 7.0) and large bead residues were filtered (0.4 mm) to generate 10% (wt/vol) slurries for further fermentation, and another portion of each sample was stored at −80°C for further study.

### *In vitro* Fermentation

Batch culture fermentation was carried out as described by Li et al. (2020). Briefly, the basal culture medium VI is composed of yeast extract (4.5 g/L), tryptone (3.0 g/L), peptone (3.0 g/L), KH_2_PO_4_ (0.4 g/L), NaCl (4.5 g/L), KCl (2.5 g/L), MgCl_2_.6H_2_O (0.45 g/L), CaCl_2_.6H_2_O (0.2 g/L), bile salts No. 3 (0.4 g/L), cysteine hydrochloride (0.8 g/L), hemin (0.05 g/L), and Tween 80 (1 mL/L). The medium was adjusted to pH 6.5, and 2 mL trace element was added before autoclaving. To assess the fermentation of EPS-m2 in HGM, 8.0 g/L starch or EPS-m2 was added as the sole carbon source. Each vessel was inoculated with 4.5 mL basal culture media and 0.5 mL of fresh slurry (total volume: 20 mL). EPS-m2 or starch was added to each vessel, and fermentation was run in an anaerobic chamber (anaerobic workstation AW 500, Electrotek Ltd., United Kingdom). An extra vessel with no added carbohydrate source (referred to here as the VI group) was also included as a control. A 1-mL fermentation broth was collected after 24, 48, and 72 h of fermentation for DNA extraction, bacterial community and SCFA detection.

### Thin-Layer Chromatography

To assess the degradation of EPS-m2 by HGM *in vitro*, thin-layer chromatography (TLC) was selected in this study. Briefly, 0.2 μL samples were taken from the supernatants of fermentation broth and spotted on pre-coated silica gel-60 TLC aluminum plates (Merck, Germany). For plate developing, plates were saturated in a solution of formic acid: n-butanol: water (6:4:1, v/v/v) for 1 h. After reaching a migration distance of 160 mm, the plates were dried out and visualized with an orcinol-sulfuric acid spray reagent.

### DNA Extraction

A QIAamp DNA Stool Mini Kit (QIAGEN, Germany) was selected for total DNA isolation according to the manufacturer’s instructions. The DNA concentration and integrity were evaluated by using a Nanodrop ND-2000 (NanoDrop Technologies, United States) and a 1.0% (w/v) gel electrophoresis. The extracted DNA samples were stored at −20°C and used as template in PCR amplification.

### 16S rRNA Gene High-Throughput Sequencing and Analysis

To assess the microbial diversity of fermentation samples with EPS-m2, 16S rRNA gene amplicon sequencing was performed. The hypervariable region of 16S rRNA gene V3-V4 was amplified with the universal primers 338F (5′-ACTCCTACG GGAGGCAGCA-3′) and 806R (5′-GGACTACHVGGGTWTCTAAT-3′). An Illumina MiSeq 300PE system was used to sequence these samples by Majorbio Bio-Pharm Technology Co., Ltd. (Shanghai, China). Subsequent bioinformatics analysis was processed as described by Serafini et al. (2014). The raw data were deposited in the NCBI database as PRJNA644207. Basic information of 16S rRNA gene high-throughput sequencing is given in Supplementary Table S1.

### Determination of SCFA Concentration

Fecal SCFA concentrations were assessed by gas chromatography (GC) according to previously published methods (Yang et al., 2014). In brief, samples collected from the batch culture fermentation (1 mL) were diluted with 0.2 mL of 25% (w/v) metaphosphoric acid. After centrifugation at 4,000 g for 20 min at room temperature, the supernatant was filter-sterilized (0.22 μm) and the SCFA concentrations in the supernatant were then determined using GC (Shimadzu, GC-2010 Plus, Japan) equipped with a InertCap FFAP column (0.25 mm × 30 m × 0.25 μm). GC Solution software and the single-point internal standard method were used for data processing. Three SCFA standards were used for identification and quantification including acetic, propionic, and butyric.

### Statistical Analysis

The phylogenetic tree was computed using weighted and unweighted UniFrac metrics (Lozupone et al., 2011). Principal coordinate analysis (PCoA) plots were created using EMPeror v0.9.3-dev (Vazquez-Baeza et al., 2013). Sequences sharing 97% identity were assigned to the same operational taxonomic units (OTUs) using the Mothur program, and linear discriminant analysis (LDA) was performed on the website http://huttenhower.sph.harvard.edu/galaxy (Segata et al., 2011). All results are expressed as the mean ± standard deviation, and statistical analysis was performed by using SPSS version 20.0 software. Variance Tamhane’s T2 (M) test was used for compare the different between groups. Statistically significance was considered when *p* < 0.05.

## Results and Discussion

### Ceftriaxone Increases the Production of EPS-m2 in *E*. *coli* WM3064

**FIGURE 1:**
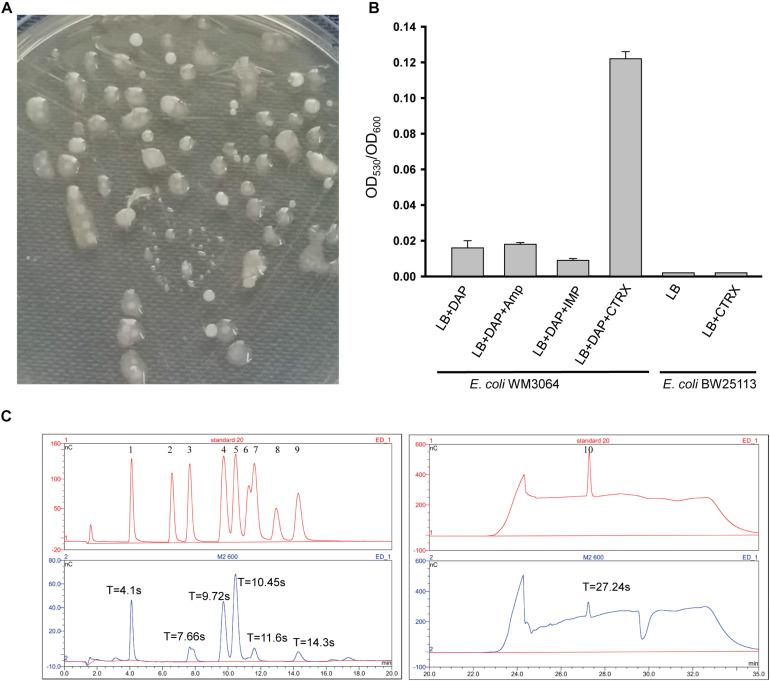
Production and monosaccharide composition of EPS-m2. **(A)** Mucoid colonies of EPS-m2-producing bacterial on LB plates containing 0.3 mM DAP. **(B)** EPS-m2 yields in LB medium with different antibiotic agents were determined by the ratio of OD_530_ to OD_600_. **(C)** The monosaccharide composition of EPS-m2 was measured by using the DIONEX ICS 5000 system. The picture of “standard 20” represents the 10 standard monosaccharides. 1–10 represents the peaks of the standards L-fucose, L-rhamnose, L-arabinose, D-galactose/N-acetyl glucosamine, D-glucose, D-mannose, D-xylose, D-fructose, D-ribose, and D- glucuronic acid, respectively. “T” represents the retention time for absorption peaks. Amp, ampicillin; IMP, imipenem; CTRX, ceftriaxone. The concentrations of these three antibiotics used in this study were all 0.5 μg/mL.

*Escherichia coli* WM3064 can produce exopolysaccharide, and EPS was designated as EPS-m2 (Figure 1A), but the yield is low and not consistent. To determine the condition of EPS-m2 production in *E*. *coli* WM3064, three antibiotic agents (ceftriaxone, ampicillin, and imipenem) were used. The results illustrated that adding 0.5 μg/mL ceftriaxone could significantly increase the yields of EPS-m2 in *E*. *coli* WM3064 (Figure 1B). Furthermore, we confirmed that 0.25 and 0.125 μg/mL of ceftriaxone could not increase the production of EPS-m2, and the strain could not sustain growth at 1.0 μg/mL of ceftriaxone. Meanwhile, another two antibiotics, ampicillin and imipenem, were evaluated for EPS-m2 production, and the results showed that these two antibiotics could not induce the production of EPS-m2 (Figure 1B).

In this study, we isolated an *E*. *coli* WM3064 mutant from LB plates containing 0.5 μg/mL ceftriaxone. Then, the mutant was purified with repeated restreaking and the purity was confirmed by the uniformity of cell morphology and 16S rRNA based DNA sequencing. Moreover, the yields of EPS-m2 remained stable in *E. coli* WM3064 mutants with important implications for inheritance. Meanwhile, the MIC value of ceftriaxone for the mutants was 2.0 μg/mL compared with 1.0 μg/mL for the wild type. These results indicate that *E*. *coli* WM3064 producing EPS-m2 undergo genetic mutations in favor of the production of EPS-m2 in their genomes under the treatment of ceftriaxone, and the mechanism remains to be explored in future studies. Our results follow previous studies reporting the reduced sensitivity to ceftriaxone due to enhanced exopolysaccharide production (Hoff and Kristich, 2016). Moreover, we analyzed the genotype of WM3064 (Dehio and Meyer, 1997), and no ceftriaxone-resistance determinants were found. The previous studies have confirmed that *Enterococcus faecalis* is increasingly sensitive to ceftriaxone due to reduced dTDP-glucose synthesis that is necessary for the biosynthesis of exopolysaccharide (Hoff and Kristich, 2016). Thus, we propose that ceftriaxone may induce EPS-m2 production and then enhance the intrinsic ceftriaxone resistance of *E*. *coli* WM3064 by regulating the dTDP-glucose synthesis. Still, the authentic mechanisms remain to be explored. Furthermore, to examine the effects of ceftriaxone on the EPS-m2 production of *E*. *coli* K-12 BW25113, the same experiments were conducted, and the results showed that ceftriaxone could not induce the production of EPS-m2 in BW25113 (Figure 1B). Thus, the characteristics of EPS-m2 production are a unique property for *E*. *coli* WM3064.

EPS-m2 produced by *E*. *coli* WM3064 was chemically characterized, and the results showed that carbohydrates, proteins, and uronic acids in the EPS-m2 accounted for 78.6 ± 0.1%, 4.38 ± 0.11%, and 3.86 ± 0.09%, respectively. The monosaccharide composition analysis of EPS-m2 was performed using a DIONEX238 ICS 5000 High Purity Capillary ion chromatography system. Ten monosaccharide standards were used as a reference for monosaccharide retention time calculation. As shown in Figure 1C, EPS-m2 consisted of seven monosaccharides including glucuronic acid, glucose, fucose, galactose/N-acetyl glucosamine, arabinose, xylose, and ribose among the standard monosaccharides, and the molar ratio of these monosaccharides was approximately 77:44:29:28:2:1:1. Moreover, d-glucuronic acid was the most abundant monosaccharide in EPS-m2, followed by D-galactose/N-acetyl glucosamine.

It has been reported that *E*. *coli* can produce various EPSs, including CA, PGA, cellulose, and EPS-RB (Beloin et al., 2008; Li et al., 2020). Compared with the known *E. coli* EPSs, the monosaccharide composition of EPS-m2 is more similar to EPS-RB, but the molecular ratio is different. It has been suggested that composition and structure of polysaccharides affected their physicochemical properties very much (Joyce et al., 2003). However, there is a lack of systematic investigation for whether such structural variations have effects on biological functions.

### Degradation of EPS-m2 by Human Gut Microbiota *in vitro*

**FIGURE 2:**
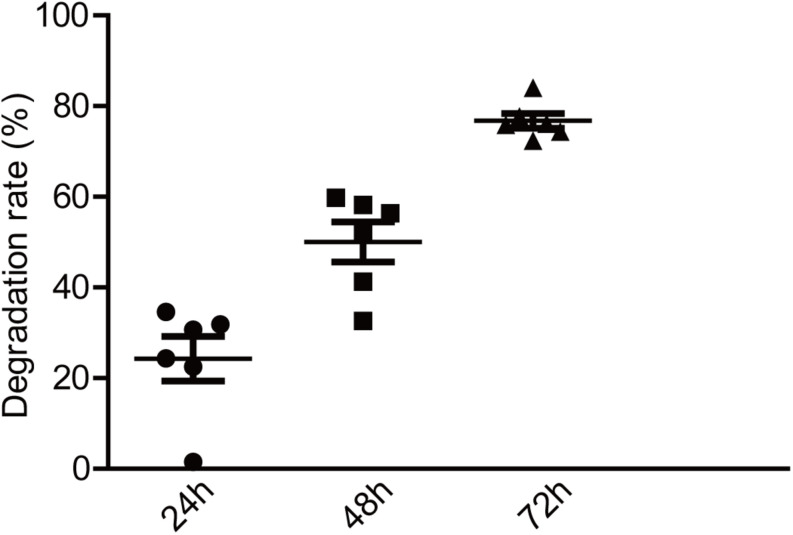
Degradation of EPS-m2 by HGM *in vitro*. IntDen of each dot in TLC plates was measured using ImageJ software (https://imagej.nih.gov/ij; National Institutes of Health, Bethesda, MD, United States). The degradation rates of EPS-m2 by HGM were then calculated.

Numerous studies have shown that exopolysaccharides exert their biological activities mainly through a degradation reaction by intestinal microbiota (Hamaker and Tuncil, 2014). To investigate the potential relationship between EPS-m2 and HGM, the degradation of EPS-m2 was conducted with varying initial inocula and fermentation times. The EPS-m2 mixed with VI-culture was used as a control. In this study, TLC was used to analyze the degradation of EPS-m2 by HGM during *in vitro* fermentation (Supplementary Figure S1). To quantitatively assess the degradability of EPS-m2 by gut microbiota, the gray value was extracted from the TLC results. As shown in Figure 2, only ∼20% of EPS-m2 was degraded after 24 h fermentation, but up to ∼50 and 77% of EPS-m2 was degraded after 48 and 72 h fermentation, respectively. Gut microbial populations are important mediators of fermentation of dietary polysaccharides by producing an arsenal of enzymes that degrade polysaccharides that cannot be hydrolyzed by host enzymes (Kaoutari et al., 2013). The composition of the human fecal microbiota responds to dietary carbohydrate intake and cause individual discrepancies in gut microbiota community. Accordingly, the inter-individual variation in microbiota composition can strongly affect individual intake dietary carbohydrate (Walker et al., 2011). In this study, the bacterial community diversities in all the fecal samples were determined by bacterial 16S rRNA gene sequencing, and *Bacteroidetes* and *Firmicutes* were found to be the most abundant in all human fecal microbiota (Supplementary Figure S2). It is reported that the species *Bacteroides thetaiotaomicron* encodes a large number of carbohydrate degrading enzymes and has the ability to switch diet carbohydrate to digestible carbohydrates (Xu et al., 2003; Sonnenburg et al., 2005). Perhaps not surprisingly, EPS-m2 could be degraded by HGM. Thus, we propose that, like EPS-RB, the degradation of EPS-m2 depends on the diversity and abundance of gut microbiota.

### EPS-m2 Regulated the Composition of Human Gut Microbiota

The gut microbiota use a variety of strategies to process and scavenge polysaccharides, and the degradation of polysaccharides can also modulate the gut microbiota population and metabolic functions of microbial communities through cross-feeding networks where metabolites are exchanged (Chung et al., 2016; Zhang et al., 2020). To evaluate the impacts of EPS-m2 on human fecal microbiota alteration, the gut microbiota composition of the control and EPS-m2 groups were measured using 16S rRNA gene sequencing. For 6 original fecal samples, 215,800 high-quality reads were obtained; for 24 fermented samples, 1,357,221 high-quality reads were obtained (Supplementary Table S1).

At a 97% similarity level, 5,658 OTUs were identified in all samples and an average of 135 OTUs for each sample (Supplementary Table S1). The Ace index, chao index, Shannon diversity index, and Simpson diversity index were selected and used to estimate the richness and diversity of gut microbiota, and no remarkable differences among groups were found (Supplementary Table S1). As shown in Supplementary Figure S3, the rarefaction curves and Shannon-Wiener curves indicated that sequencing depth was sufficient to yield a stable estimate of the phylotype richness (Supplementary Figure S3). The Rank-abundance distribution curves indicate the abundance and distribution of the various groups was evenness (Supplementary Figure S3).

**FIGURE 3:**
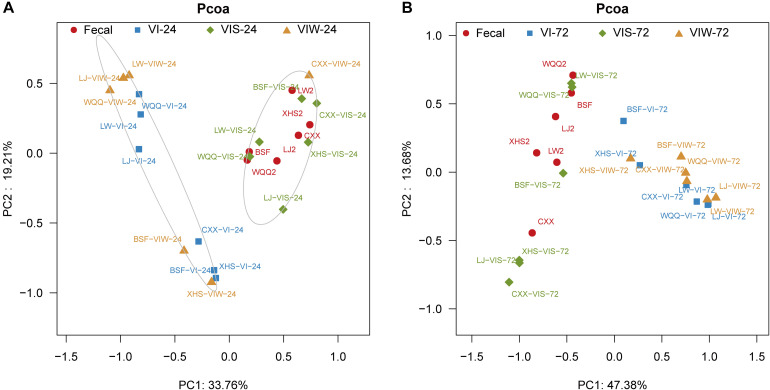
PCoA analysis of the microbial composition of samples. “Fecal” represents original fecal samples. “VIW” and “VIS” represent fermented samples adding EPS-m2 and starch, respectively. “24” and “48” represents the fermentation time of 24 h **(A)** and 48 h **(B)**. VI represents the fermented samples using VI media.

**FIGURE 4:**
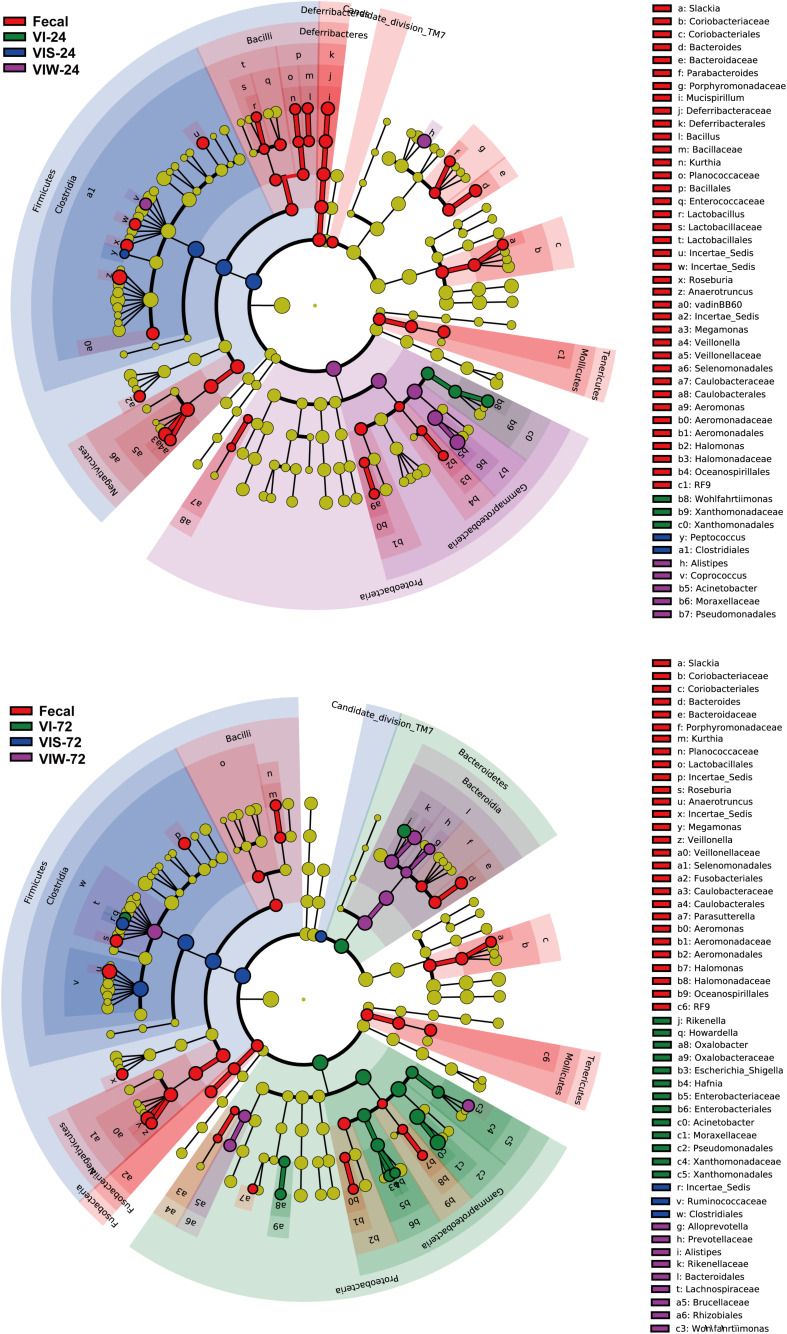
Identification of significantly different bacteria using LEfSe. “Fecal” represents the volunteers’ original fecal samples. “VIW” and “VIS” represent fermented samples after adding EPS-m2 and starch, respectively. “24” and “48” represents the fermentation time.

β-diversity was determined in order to characterize the changes between each group on the gut microbiota structure. As the results indicated that the gut microbial composition and diversity of the VIW group was significantly different from that of the control group (Supplementary Figure S2). At the phylum level, *Firmicutes*, *Bacteroidetes*, *Proteobacteria*, and *Actinobacteria* were dominated in the gut microbiota in all groups. After 72 h of EPS-m2 fermentation, the relative abundance of *Firmicutes* was reduced and *Proteobacteria* was greater than that of the control group (Supplementary Figure S2). PCoA analysis showed that the gut microbiota of VIW group differed from the control group, and VIW group were clustered together at both points in time (Figure 3). The distance between the VI and VIW (EPS-m2) groups were closer than the control group, and clear separation formed between the VIW group and the control group in all the volunteers after fermentation of 24 and 72 h (ANOVA, *p* < 0.001) (Figure 3). Overall, the bacteria community profiles of the VIW group were similar with the VI group, but different with the control group. These findings indicate that EPS-m2 have a putative regulatory effect on human fecal microbiota. To identify the specific bacteria phylotypes that differentially altered between the VIW (EPS-m2) group and the controls, the linear discriminant analysis effect size (LEfSe) method was used. As shown in Figure 4, the relative abundance of the dominant genera in original inocula and the fermented groups were remarkable different (Figure 4). The relative abundance of *Prevotella* in the No. 3, No. 5, and No. 6 fecal samples was significantly higher than that of other samples, whereas the relative abundance of *Bacteroides* in fecal samples No. 1, No. 2, and No. 4 was higher than other fecal samples (Supplementary Figure S2). Moreover, the profile of enriched bacteria was analyzed at genus level using LEfSe analysis. The relative abundance of *Alistipes*, *Acinetobacter*, *Alloprevotella*, *Howardella*, and *Oxalobacter* were enriched by EPS-m2 compared with another two groups (Figure 4). Previous studies have suggested that *Alistipes* are involved in the fermentation of polysaccharides; for example, *Alistipes putredinis* could degrade fiber and glucosinolates (Li et al., 2009) and *Alistipes finegoldii* was involved in the metabolism of glycans (Nihira et al., 2013). In this study, the genus *Alistipes* was significantly increased after EPS-m2 treatment at 24 and 72 h. The more abundant *Alistipes* might be linked to EPS-m2 degradation in the human intestine. It has been reported that *Acinetobacter* can degrade and use benzyl alcohol, black liquor, and kraft lignin as carbon sources (Lopretti et al., 1993). However, there is no report that *Acinetobacter* was related to degrading bacterial polysaccharides. Fermentation *in vitro* revealed that fucoidan modulates gut microbiota and an increased abundance of *Alloprevotella* (Liu et al., 2018). Also, the abundance of genera *Alloprevotella* increased during *in vitro* fermentation of bee-collected pollen of Chinese wolfberry (Zhou et al., 2018). Recently, studies have revealed that the high-fiber group is associated with increased abundances of *Oxalobacter* compared with the low-fiber group (Adamberg et al., 2020). However, there is no report that *Howardella* is enriched by EPSs before. It has been well-characterized that different resources of polysaccharides have different chemical structures, which might influence the profiles of populations and metabolites of bacterial groups. Although EPS-m2 and EPS-RB share a similar monosaccharide composition, their molecular proportion is significantly different and, given their different chemical structures, they are proposed to exert diverse functions. As expected, the fermentation of EPS-m2 enriched the microbiota of *Alistipes*, *Acinetobacter*, *Alloprevotella*, *Howardella*, and *Oxalobacter* compared with *Collinsella*, *Butyricimonas*, and *Hafnia* for EPS-RB (Li et al., 2020). Thus, these interactions suggest that these bacterial species (especially members of *Alloprevotella*) within the colonic microbiota may serve as potential microbiota markers for EPS-m2. These results also indicate that EPS-m2 affects the modification of HGM and has putative clinical applications. Future studies must be performed to elucidate the functions of EPS-m2 that was produced by *E*. *coli* WM3064.

### Detection the Variable of SCFAs Concentration

**FIGURE 5:**
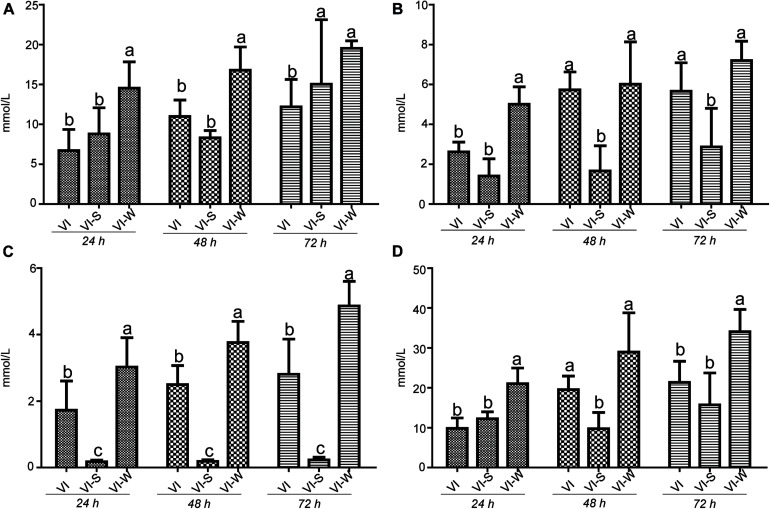
Influence of EPS-m2 fermentation on SCFA production. The concentration of **(A)** acetic acid, **(B)** propionic acid, **(C)** butyric acid, and **(D)** and total SCFAs were measured using gas chromatography, and total SCFA represents the summary of acetic acid, propionic acid and butyric acid. “VIW,” “VIS,” and “VI” represent samples collected after EPS-m2 fermentation, starch fermentation, and no carbohydrate added, respectively. Significant differences between treatments were labeled using different normal letters. Triplicate measurements were performed for each sample in this study.

It has long been recognized that dietary polysaccharides affect not only the gut microbiota but also the levels of SCFAs in the gut tract. SCFAs have wide-ranging impacts on various aspects of host physiology, such as host metabolism, immune system, and cell proliferation (Hu et al., 2018). Moreover, the production of SCFA could be regulated by some factors including the soluble fermentable carbohydrates. To test the potential capacity of EPS-m2 to produce SCFA, we first examined the effects of EPS-m2 and starch on the SCFAs levels (acetic, propionic, butyric, and total SCFAs). The results showed that the levels of SCFAs, including acetic, propionic, butyric, and total SCFAs among all groups did not change significantly after 24 h of fermentation (Figure 5). However, the concentrations of acetic, propionic, butyric, and total SCFAs became significantly higher than in the VI group after fermenting EPS-m2 for 48 and 72 h (Figure 5). Interestingly, the gut microbiota analyses revealed that EPS-m2 levels were significantly higher the relative abundances of some strains belonging to the genera *Alistipes*, *Acinetobacter*, *Alloprevotella*, *Howardella*, and *Oxalobacter* (Figure 4). Thus, it is likely that the higher fecal SCFA concentrations in the VIW group may have resulted from an increase in the gut SCFA-producing bacterial genera, such as *Alistipes* and *Alloprevotella* (Zhao et al., 2013; Shang et al., 2017). Although the fermentation of EPS-m2 also increased the production of total SCFAs, the levels of acetic, propionic, butyric, and total SCFAs with EPS-m2 supplementation were lower than with EPS-RB supplementation (Li et al., 2020) during the fermentation progress. The differences in the levels of SCFAs between VIW and VIR (fermented samples adding EPS-RB) could be due to their differences in the chemical structure of EPSs and the functions on modulating the gut microbial communities. Consistent with these results, previous studies also showed that the type of polysaccharides has dramatic effects on the composition of the intestinal microbiota and consequently on the SCFAs production (LeBlanc et al., 2017). It has been reported that gut SCFAs can be absorbed by the host and contribute to energy generation, especially butyrate (LeBlanc et al., 2017). Moreover, HGM benefits from the metabolism of polysaccharides. In this study, EPS-m2 fermentation modulated the gut microbiota and enhanced the SCFAs production *in vitro*. Thus, our observations on SCFAs and community composition changes suggest that EPS-m2 could be used as a potential probiotic, and further studies are required to identify the mechanism involved in the regulation of the gut microbiota and increased SCFA levels.

## Conclusion

This study presents an initial report on the EPS-m2 production in *E*. *coli* WM3064 and *in vitro* fermentation of EPS-m2 by HGM. It seems evident that conditions associated with life outside of the *E*. *coli* WM3064, such as antibiotics induce EPS production. The present findings provide a strategy to screening new EPS and enhancing EPS production. Our work not only confirms that EPS-m2 may be a new exopolysaccharide from *E*. *coli* WM3064 but also demonstrates the modulation of EPS-m2 on the gut microbiota community. These results reveal potential health-promoting functions of EPS-m2. Further investigations will require to determine if the observed effects can be expected from human diet modifications.

## Data Availability Statement

All datasets generated for this study are publicly available. This data can be found here: https://www.ncbi.nlm.nih.gov/bioproject/?term=PRJNA644207.

## Author Contributions

YY and BL conceptualized and designed the project, did supervision and visualization, wrote, reviewed, and edited of original draft. BL, HC, LC, DC, and YY did investigation, data curation, and data analysis. All authors contributed to the article and approved the submitted version.

## Conflict of Interest

The authors declare that the research was conducted in the absence of any commercial or financial relationships that could be construed as a potential conflict of interest.
