# Supplementary material for: Escherichia coli Exopolysaccharides Induced by Ceftriaxone Regulated Human Gut Microbiota in vitro
Source: Front Microbiol. 2021 Feb 16;12:634204. doi: 10.3389/fmicb.2021.634204 (PMC7928337; doi:10.3389/fmicb.2021.634204)
Supplement: Supplementary file 1 [file Data_Sheet_1.docx]

**Supplementary File**

***Escherichia coli* exopolysaccharides induced by ceftriaxone regulated human gut microbiota in vitro**

Baiyuan Li^1^, Huahai Chen^1^, Linyan Cao^1^, Yunfei Hu^1^, Dan Chen^1^, Yeshi Yin^1,2^*

^1^Key Laboratory of Comprehensive Utilization of Advantage Plants Resources in Hunan South, College of Chemistry and Bioengineering, Hunan University of Science and Engineering, Yongzhou, Hunan, China

^2^State Key Laboratory of Breeding Base for Zhejiang Sustainable Pest and Disease Control, Institute of Plant Protection and Microbiology, Zhejiang Academy of Agricultural Sciences, Hangzhou, Zhejiang, China

***Corresponding author**:

Yeshi Yin, Tel: (86746)-2382989; Email: yinyeshi@126.com.

**Keywords:** EPS-m2, ceftriaxone, gut microbiota, 16S rRNA gene sequencing, SCFA

**Figure S1. Degradation effects of human and mouse gut microbiota in vitro on EPS-m2 detected by TLC.** A volume of 0.2 μL of each sample collected 24 and 72 h after fermentation under anaerobic conditions was used for TLC analysis. R represents the origin of EPS-m2; NO.1- NO.6 represent the fecal sample of the six volunteers (NO.1, BSF; NO.2, CXX; NO.3, LJ; NO.4, LW; NO.5, WQQ; NO.6, XHS) inoculated for the fermentation experiment.

**
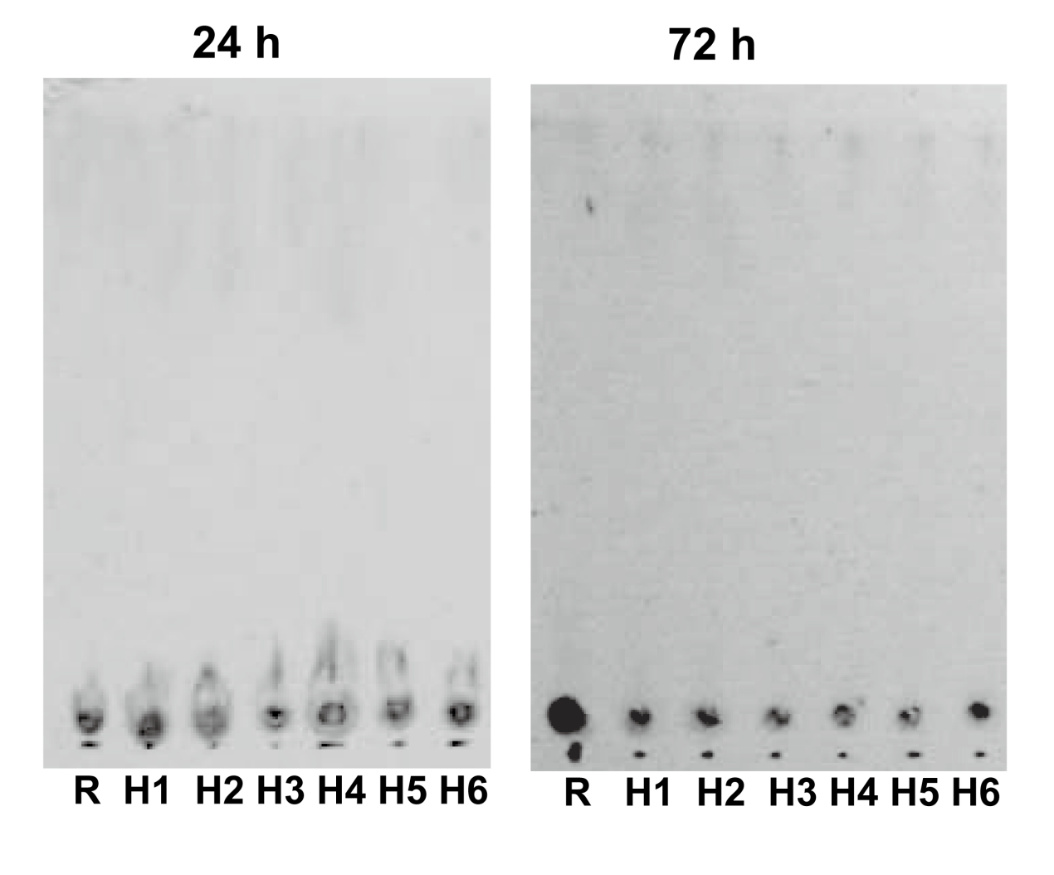
**

**Figure S2. Bacterial community abundance at the phylum level.** NO.1, NO.2, NO.3, NO.4, NO.5 and NO.6 represents the original fecal samples. VIS and VIW represent samples fermented in cultural media VI with EPS-m2 and starch added as substrate, respectively. VI represents samples fermented in basic cultural medium with no carbohydrate added.

**
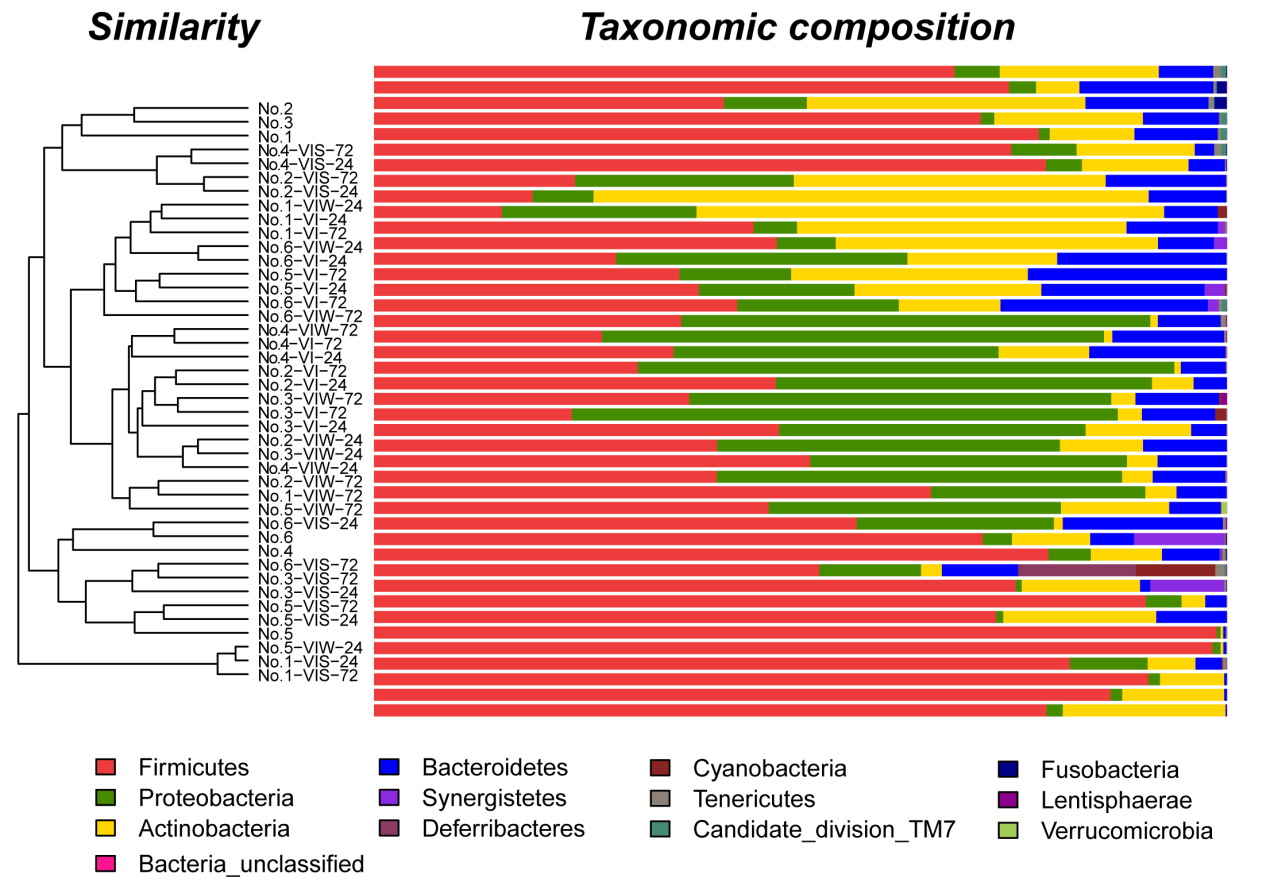
**

**Figure S3. Basic analysis of sequenced data. (**A) Rarefaction curves of sequenced samples. (B) The Shannon-Wiener curves of the sequenced samples. (C) Specaccum analysis of the sequenced samples. (D) Rank-abundance distribution curve of the sequenced samples.

**
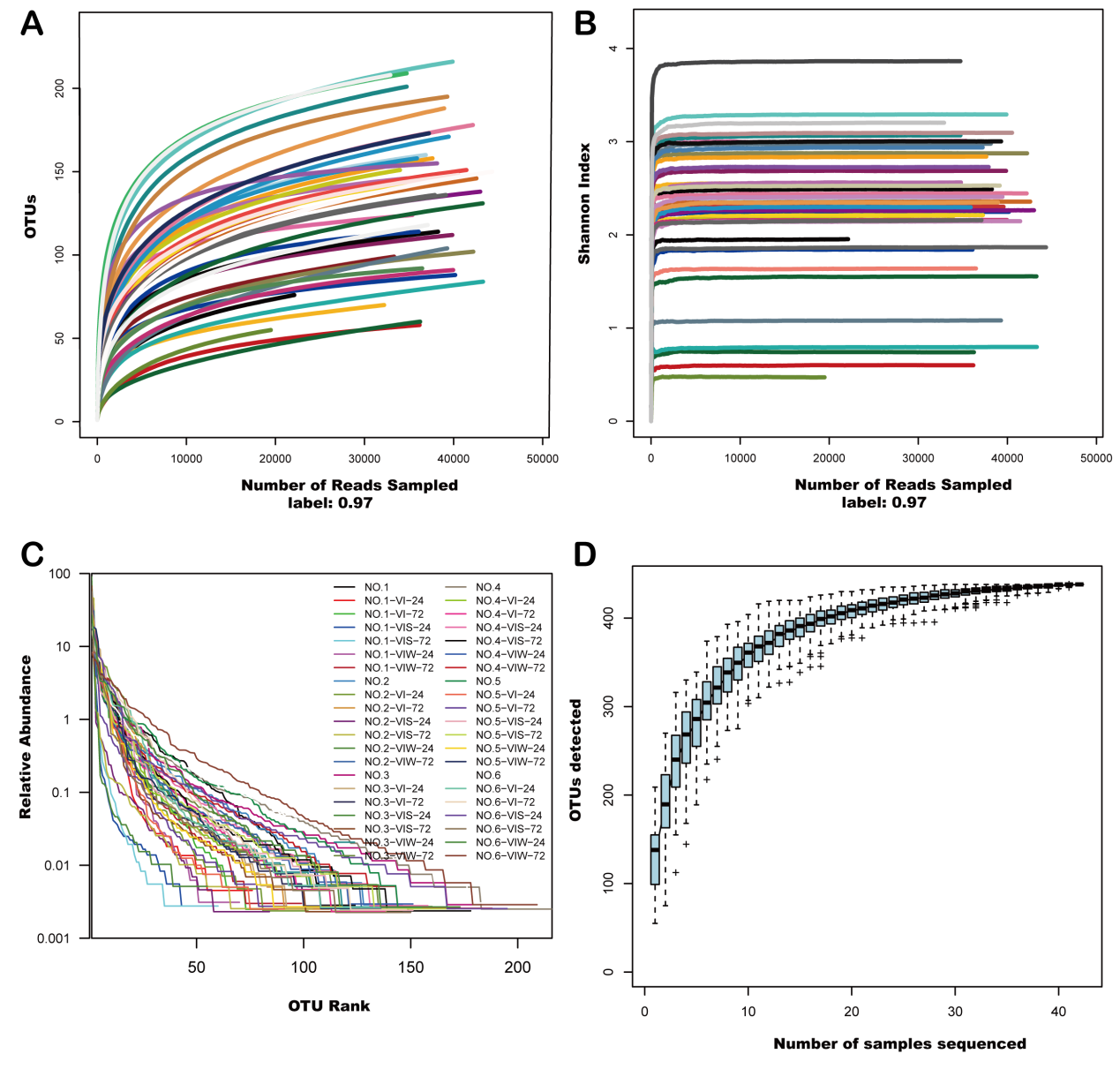
**

**Table S1. Basic information on the volunteers.**

| Sample ID | Age (years) | Sex | Reads | OUT（0.97） | Ace | Chao | Shannon | Simpson | Coverage |
| --- | --- | --- | --- | --- | --- | --- | --- | --- | --- |
| NO.1 | 24 | Male | 35373 | 124 | 140.124331 | 169.333333 | 3.007848 | 0.119416 | 0.999519 |
| NO.1-VI-24 |  |  | 22113 | 76 | 97.942907 | 114.5 | 1.953647 | 0.255212 | 0.999005 |
| NO.1-VI-72 |  |  | 40174 | 88 | 103.190262 | 100 | 2.247625 | 0.178387 | 0.999602 |
| NO.1-VIS-24 |  |  | 36172 | 58 | 71.707301 | 73 | 0.600507 | 0.743247 | 0.999558 |
| NO.1-VIS-72 |  |  | 36238 | 60 | 154.517391 | 96.111111 | 0.740709 | 0.633751 | 0.999283 |
| NO.1-VIW-24 |  |  | 32196 | 70 | 119.13474 | 108 | 2.557503 | 0.111722 | 0.999379 |
| NO.1-VIW-72 |  |  | 33318 | 99 | 155.037561 | 149.142857 | 2.343079 | 0.164113 | 0.99919 |
| NO.2 | 23 | Female | 38138 | 155 | 161.204708 | 162.333333 | 2.978429 | 0.133208 | 0.999685 |
| NO.2-VI-24 |  |  | 39948 | 91 | 104.559942 | 100.714286 | 2.158922 | 0.179885 | 0.999574 |
| NO.2-VI-72 |  |  | 36469 | 92 | 101.658351 | 99.583333 | 1.63944 | 0.321971 | 0.999616 |
| NO.2-VIS-24 |  |  | 41437 | 151 | 173.798163 | 187.25 | 2.146248 | 0.280261 | 0.999276 |
| NO.2-VIS-72 |  |  | 39445 | 171 | 207.351841 | 212.166667 | 2.40759 | 0.202319 | 0.999011 |
| NO.2-VIW-24 |  |  | 37085 | 118 | 153.772724 | 176.666667 | 2.156891 | 0.198981 | 0.99911 |
| NO.2-VIW-72 |  |  | 39133 | 136 | 160.53596 | 167.230769 | 2.528801 | 0.127613 | 0.999259 |
| NO.3 | 25 | Female | 34725 | 201 | 228.620805 | 234.055556 | 3.067284 | 0.099218 | 0.998992 |
| NO.3-VI-24 |  |  | 38254 | 114 | 135.250364 | 133.461538 | 2.489327 | 0.151069 | 0.999399 |
| NO.3-VI-72 |  |  | 36080 | 114 | 123.09999 | 123.545455 | 1.841329 | 0.365699 | 0.999584 |
| NO.3-VIS-24 |  |  | 39565 | 149 | 179.643647 | 182 | 2.301339 | 0.187141 | 0.999166 |
| NO.3-VIS-72 |  |  | 43261 | 131 | 161.321081 | 160.0625 | 1.554451 | 0.462819 | 0.999283 |
| NO.3-VIW-24 |  |  | 37251 | 145 | 165.817615 | 168.625 | 2.211573 | 0.221903 | 0.999248 |
| NO.3-VIW-72 |  |  | 36870 | 160 | 179.540956 | 174.130435 | 2.369926 | 0.234978 | 0.999295 |
| NO.4 | 38 | Male | 39888 | 216 | 245.273596 | 240.391304 | 3.291841 | 0.071784 | 0.999148 |
| NO.4-VI-24 |  |  | 42561 | 146 | 175.600894 | 202.1 | 2.356672 | 0.170899 | 0.999201 |
| NO.4-VI-72 |  |  | 42984 | 138 | 161.495585 | 157.117647 | 2.263772 | 0.244148 | 0.999395 |
| NO.4-VIS-24 |  |  | 35880 | 158 | 179.743084 | 181.625 | 2.295957 | 0.268522 | 0.99922 |
| NO.4-VIS-72 |  |  | 42161 | 178 | 212.939786 | 223.882353 | 2.444465 | 0.208725 | 0.999051 |
| NO.4-VIW-24 |  |  | 33973 | 151 | 182.071541 | 182 | 2.165153 | 0.25437 | 0.999088 |
| NO.4-VIW-72 |  |  | 38936 | 188 | 240.912381 | 254 | 2.347703 | 0.267238 | 0.998844 |
| NO.5 | 25 | Female | 34785 | 146 | 160.249011 | 158.214286 | 2.562437 | 0.169576 | 0.999454 |
| NO.5-VI-24 |  |  | 42207 | 102 | 121.548997 | 119.769231 | 2.875067 | 0.082193 | 0.999479 |
| NO.5-VI-72 |  |  | 39850 | 112 | 135.276276 | 137.666667 | 2.68647 | 0.129304 | 0.999448 |
| NO.5-VIS-24 |  |  | 43300 | 84 | 154.451136 | 127.875 | 0.796992 | 0.667599 | 0.999376 |
| NO.5-VIS-72 |  |  | 39274 | 104 | 214.169883 | 165.75 | 1.081392 | 0.438467 | 0.999007 |
| NO.5-VIW-24 |  |  | 19471 | 55 | 72.802721 | 67.363636 | 0.471188 | 0.817875 | 0.999127 |
| NO.5-VIW-72 |  |  | 37626 | 158 | 189.677277 | 185.555556 | 2.839433 | 0.111103 | 0.99915 |
| NO.6 | 24 | Male | 32891 | 208 | 233.478989 | 237.176471 | 3.202983 | 0.08815 | 0.999027 |
| NO.6-VI-24 |  |  | 37893 | 136 | 164.853247 | 161.375 | 2.728157 | 0.130864 | 0.999235 |
| NO.6-VI-72 |  |  | 40521 | 148 | 170.948922 | 167.5 | 3.095679 | 0.077546 | 0.999334 |
| NO.6-VIS-24 |  |  | 39293 | 195 | 215.018338 | 217.555556 | 3.002897 | 0.088369 | 0.999262 |
| NO.6-VIS-72 |  |  | 44335 | 150 | 207.822201 | 180 | 1.866374 | 0.33673 | 0.999188 |
| NO.6-VIW-24 |  |  | 37222 | 173 | 209.82586 | 208.15 | 2.938166 | 0.113318 | 0.998979 |
| NO.6-VIW-72 |  |  | 34725 | 209 | 232.190264 | 242.214286 | 3.863426 | 0.033651 | 0.999107 |
